# Age Impairs Soluble Guanylyl Cyclase Function in Mouse Mesenteric Arteries

**DOI:** 10.3390/ijms222111412

**Published:** 2021-10-22

**Authors:** Cheng Zhong, Minze Xu, Sengül Boral, Holger Summer, Falk-Bach Lichtenberger, Cem Erdoğan, Maik Gollasch, Stefan Golz, Pontus B. Persson, Johanna Schleifenbaum, Andreas Patzak, Pratik H. Khedkar

**Affiliations:** 1 Institute of Vegetative Physiology, Charité—Universitätsmedizin Berlin, Corporate Member of Freie Universität Berlin and Humboldt-Universität zu Berlin, Charitéplatz 1, 10117 Berlin, Germany; cheng.zhong@charite.de (C.Z.); minze.xu@charite.de (M.X.); falk.lichtenberger@charite.de (F.-B.L.); cem.erdogan@charite.de (C.E.); pontus.persson@charite.de (P.B.P.); johanna.schleifenbaum@charite.de (J.S.); pratik.khedkar@charite.de (P.H.K.); 2 Institute of Pathology, Charité—Universitätsmedizin Berlin, Corporate Member of Freie Universität Berlin and Humboldt-Universität zu Berlin, Charitéplatz 1, 10117 Berlin, Germany; senguel.boral@charite.de; 3 Bayer AG, Research & Development, 42113 Wuppertal, Germany; holger.summer@bayer.com (H.S.); stefan.golz@bayer.com (S.G.); 4 Experimental and Clinical Research Center (ECRC), Charité—Universitätsmedizin Berlin, 13125 Berlin, Germany; maik.gollasch@charite.de; 5 Department of Internal and Geriatric Medicine, University of Greifswald, Geriatric Medicine, 17475 Greifswald, Germany

**Keywords:** aging, mesenteric artery, nitric oxide, soluble guanylyl cyclase, soluble guanylyl cyclase activator

## Abstract

Endothelial dysfunction (ED) comes with age, even without overt vessel damage such as that which occurs in atherosclerosis and diabetic vasculopathy. We hypothesized that aging would affect the downstream signalling of the endothelial nitric oxide (NO) system in the vascular smooth muscle (VSM). With this in mind, resistance mesenteric arteries were isolated from 13-week (juvenile) and 40-week-old (aged) mice and tested under isometric conditions using wire myography. Acetylcholine (ACh)-induced relaxation was reduced in aged as compared to juvenile vessels. Pretreatment with L-NAME, which inhibits nitrix oxide synthases (NOS), decreased ACh-mediated vasorelaxation, whereby differences in vasorelaxation between groups disappeared. Endothelium-independent vasorelaxation by the NO donor sodium nitroprusside (SNP) was similar in both groups; however, SNP bolus application (10^−6^ mol L^−1^) as well as soluble guanylyl cyclase (sGC) activation by runcaciguat (10^−6^ mol L^−1^) caused faster responses in juvenile vessels. This was accompanied by higher cGMP concentrations and a stronger response to the PDE5 inhibitor sildenafil in juvenile vessels. Mesenteric arteries and aortas did not reveal apparent histological differences between groups (van Gieson staining). The mRNA expression of the α1 and α2 subunits of sGC was lower in aged animals, as was PDE5 mRNA expression. In conclusion, vasorelaxation is compromised at an early age in mice even in the absence of histopathological alterations. Vascular smooth muscle sGC is a key element in aged vessel dysfunction.

## 1. Introduction

Aged arteries contribute to cardiovascular disease [1,2]. Age-related decline in arterial function includes reduced vasorelaxation and increased vasoconstriction, which compromise organ perfusion and function. The decrease in endothelium-dependent vasodilatation that comes with age is not necessarily accompanied by structural changes in the arterial wall [3]. Disturbances in the generation or breakdown of endothelium-derived vasoactive autacoids play an important role. What is typical is the attenuated response to vasodilators or to the blockade of the nitric oxide (NO) system, as shown by numerous studies in humans, rats, and mice [3,4,5,6,7]. The NO system, prostacyclin-derived metabolites, and the endothelium-derived hyperpolarizing factor (EDHF) are major components of the endothelial dilatory function.

Although all of these systems contribute to endothelial dysfunction with age, NO is the main dilatory factor in many vascular beds such as those of the kidney, the mesentery, and the retina [8,9]. NO, produced by endothelial NO synthase, diffuses into smooth muscle cells to activate its receptor, soluble guanylyl cyclase (sGC), which catalyses cyclic guanosine monophosphate (cGMP) production. cGMP activates phosphoglycerate kinase (PGK), which in turn modulates the function of various channels and enzymes resulting in reduced cytosolic calcium levels and muscular relaxation [10]. Potential mechanisms behind age-related decline in NO system functioning comprise reduced endothelial NO synthase (eNOS) expression and function on top of increased NO scavenging by reactive oxygen species (ROS) [11,12,13]. Both mechanisms diminish endothelial NO bioavailability. Furthermore, the remodelling and functional decline of vascular smooth muscle (VSM) components, eventually leading to increased vessel stiffness, affect endothelial dilatation [14].

In contrast to the widely investigated role of the endothelial compartment, there is very limited knowledge regarding NO-induced signalling in aged VSM cells. Studies do report decreased cGMP levels and reduced sGC expression in old animals as compared to young ones [15,16], which suggest the role of sGC in the age-related dysfunction of the NO system. To test the hypothesis that sGC function is reduced with age, we investigated the mesenteric arteries of juvenile (≈13-week-old) and aged mice (≈40-week-old). Aged animals, not old, were included in the study to exclude atherosclerosis and vascular remodelling, which commonly occur in old mice and markedly influence vascular function. Resistance mesenteric arteries were used, as this is a preferred model for the study of arterial function [17].

## 2. Results

### 2.1. Vessel Activity and Pre-Contraction

The activity of juvenile and adult vessels was tested using a high-potassium physiological solution (K-PSS, 123.7 mmol L^−1^ KCl, Carl Roth, Karlsruhe, Germany). Aged vessels showed a 14% reduced response to K-PSS as compared to the juvenile vessels (3.16 ± 0.12 (*n* = 48) vs. 3.66 ± 0.13 mN (*n* = 52), Mann–Whitney test, *p* < 0.01). Phenylephrine (PE, 10^−5^ mol L^−1^) was used to pre-contract the vessels for the vasorelaxation experiments. The responses to PE, which were normalized to the respective KCl-induced contraction, were similar in both groups (120.61 ± 5.64 (aged, *n* = 31) vs. 118.56 ± 7.92% (juvenile, *n* = 38), Mann–Whitney test, *p* > 0.05).

### 2.2. Aging Impairs Endothelium-Dependent Relaxation

**Figure 1:**
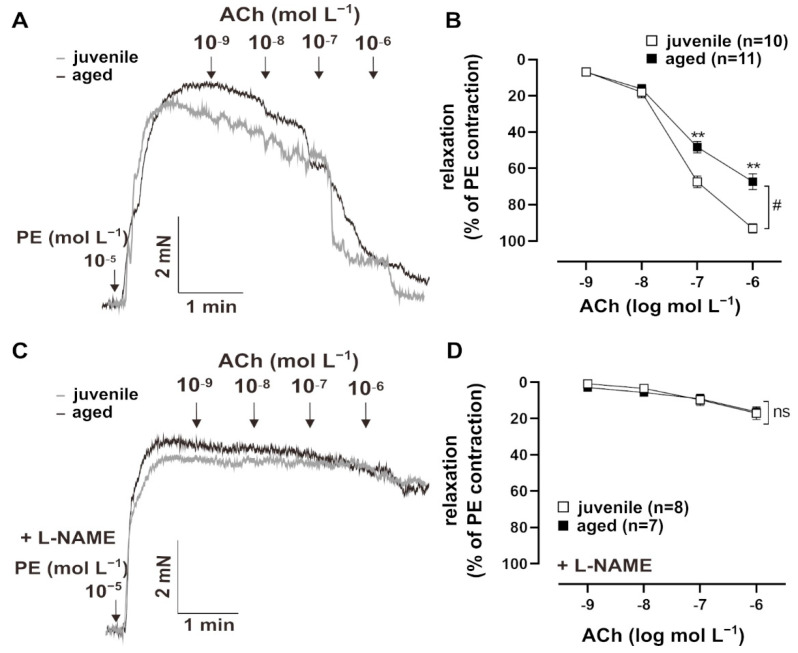
Age-related differences in relaxation to acetylcholine (ACh). (**A**) Representative traces and (**B**) concentration–response curves showing the relaxation induced by 10^−9^–10^−6^ mol L^−1^ ACh in juvenile and aged mesenteric arteries. Vessels were pre-contracted using 10^−5^ mol L^−1^ phenylephrine (PE). Juvenile vessels showed a higher maximum response to ACh compared to aged vessels (^#^ *p* < 0.05 Brunner test, ** *p* < 0.01 Mann–Whitney test). Both aged and juvenile vessels had a similar sensitivity to ACh (EC_50_: (5.60 ± 0.35) × 10^−8^ mol L^−1^ (aged), (8.03 ± 2.05) × 10^−8^ mol L^−1^ (juvenile)). (**C**) Representative traces and (**D**) concentration–response curves showing the relaxation induced by 10^−9^–10^−6^ mol L^−1^ ACh in juvenile and aged vessels. Vessels were pre-treated for 30 min with L-NAME (10^−4^ mol L^−1^, indicated as “+L-NAME”), a non-selective NOS inhibitor, and pre-contracted with PE. L-NAME largely reduced ACh-mediated relaxation in both aged and juvenile vessels. There were no differences between the aged and juvenile groups.

Vascular aging is accompanied by endothelial dysfunction [3]. To test endothelium-dependent vasorelaxation, resistance mesenteric arteries from mice were pre-contracted with PE (10^−5^ mol L^−1^) and treated with cumulatively increasing concentrations of ACh (10^−9^ to 10^−5^ mol L^−1^) (Figure 1A). Both juvenile (*n* = 10, EC_50_ = (8.03 ± 2.05) × 10^−8^ mol L^−1^) and aged (*n* = 11, EC_50_ = (5.60 ± 0.35) × 10^−8^ mol L^−1^) vessels showed concentration-dependent relaxation in response to Ach, with comparable sensitivity (EC_50_: Mann–Whitney U test, *p* > 0.05). However, aged vessels showed a significantly smaller maximum response to ACh than juvenile vessels (Figure 1B). To test the contribution of the endothelial NO system in this context, vessels were pre-treated with a non-selective inhibitor of NO synthases, *N*Μ-nitro-L-arginine methylester hydrochloride (L-NAME, 10^−4^ mol L^−1^), for 30 min [8]. The responses to ACh decreased more strongly in the juvenile group after L-NAME treatment as compared to the aged group, which resulted in similar concentration–response curves. This observation suggests that NO bioavailability is reduced in aged vessels (Figure 1C,D).

### 2.3. Endothelium-Independent Relaxation

**Figure 2:**
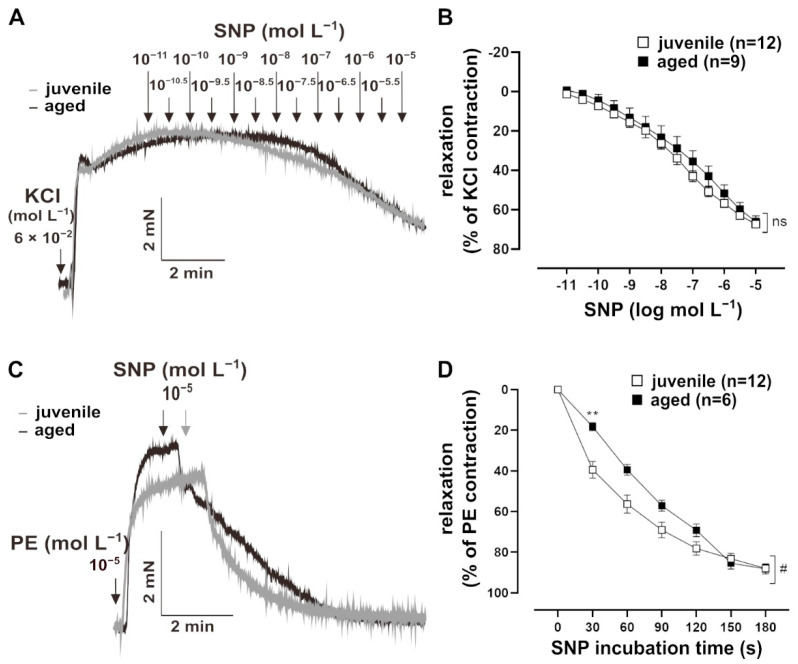
Effect of the NO donor, sodium nitroprusside (SNP), on mouse mesenteric arteries. (**A**) Representative traces and (**B**) concentration–response curves showing the relaxation induced by 10^−11^–10^−5^ mol L^−1^ SNP in juvenile and aged mesenteric arteries. Vessels were pre-treated with L-NAME (10^−4^ mol L^−1^) and pre-contracted using potassium chloride (KCl, 60 mmol L^−1^). Juvenile and aged mesenteric arteries showed similar sensitivity (EC_50_: (3.52 ± 3.34) × 10^−6^ mol L^−1^ (juvenile), (7.85 ± 4.71) × 10^−6^ mol L^−1^ (aged)) and maximum response (Brunner test, *p* >0.05, Mann–Whitney test, *p* > 0.05) to SNP. (**C**) Representative traces and (**D**) time–response curves showing relaxation induced by SNP (10^−5^ mol L^−1^) in juvenile and aged vessels over time. Vessels were pre-treated with L-NAME and pre-contracted with phenylephrine (PE). SNP caused faster relaxation in juvenile as compared to aged mesenteric arteries in the physiologically important range of up to 3 min (^#^ *p* < 0.05 Brunner test, ** *p* < 0.01 Mann–Whitney test).

To assess whether NO signalling in VSM contributes to the reduced relaxation in aged vessels, their response to the endothelium-independent vasodilator, sodium nitroprusside (SNP, 10^−11^ to 10^−5^ mol L^−1^), was measured. Vessels were pre-treated with L-NAME for 30 min, followed by a pre-contraction with 60 mmol L^−1^ KCl (Figure 2A). The sensitivity of both juvenile (*n* = 12, EC_50_ = (1.73 ± 0.46) × 10^−7^ mol L^−1^) and aged (*n* = 9, EC_50_ = (7.85 ± 4.71) × 10^−7^ mol L^−1^) vessels to SNP were similar (Mann–Whitney U test, *p* > 0.05). SNP also induced similar maximum responses in juvenile and aged vessels (Figure 2B). Remarkably, bolus application of SNP for 10 min revealed a faster relaxation in juvenile vessels as compared to aged vessels, which was most prominent during the first 2 min after NO-application (Figure 2C,D).

**Figure 3:**
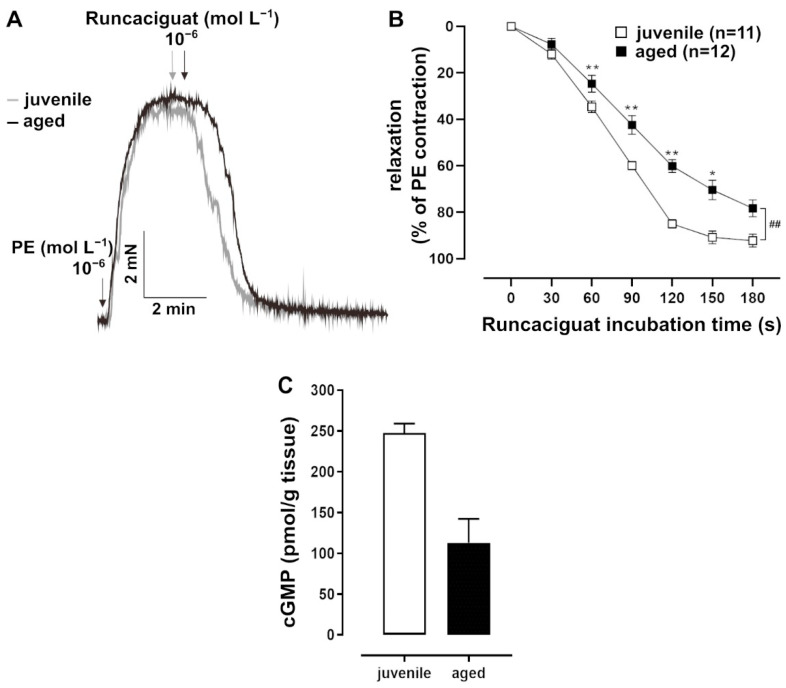
Effects of the soluble guanylate cyclase (sGC) activator runcaciguat. (**A**) Representative traces and (**B**) time–response curves showing relaxation induced by runcaciguat (10^−6^ mol L^−1^) in juvenile and aged mesenteric arteries. Vessels were pre-treated with L-NAME (10^−4^ mol L^−1^) and pre-contracted with PE. Juvenile vessels showed a significantly faster relaxation in response to runcaciguat (10^−6^ mol L^−1^) as compared to aged vessels in the physiologically important range of up to 3 min (^##^ *p* < 0.01 Brunner test, * *p* < 0.05, ** *p* < 0.01 Mann–Whitney test). (**C**) A direct cGMP ELISA was performed on tissue lysates obtained from L-NAME-treated and PE-pre-contracted arteries 2 min after bolus application of runcaciguat (10^−6^ mol L^−1^). Juvenile vessels had higher cGMP levels than aged vessels (juvenile: *n* = 2 pooled samples, each with mesenteric arterial tissue from at least 5 mice; adult: *n* = 2 pooled samples, each with tissue from 3 mice).

**Figure 4:**
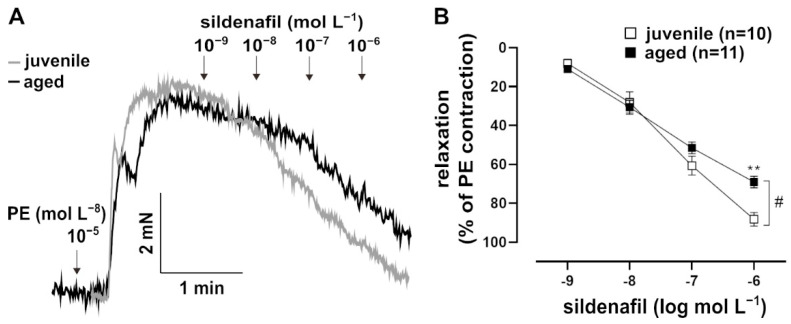
Effects of the PDE5 inhibitor, sildenafil. (**A**) Representative traces and (**B**) concentration–response curve showing the relaxation induced by 10^−9^–10^−6^ mol L^−1^ sildenafil in juvenile and aged vessels. Vessels were pre-contracted with PE. Juvenile vessels showed a higher maximum response to sildenafil as compared to aged vessels (^#^ *p* < 0.05 Brunner test, ** *p* < 0.01 Mann–Whitney test). The sensitivity of both groups to sildenafil was not significantly different (EC_50_: (5.99 ± 1.64) × 10^−8^ mol L^−1^ (juvenile), (4.93 ± 1.14) × 10^−8^ mol L^−1^ (aged)).

In addition to NO, which is a natural agonist of sGC, the artificial activator runcaciguat (BAY60-2770, Bayer AG, Wuppertal, Germany) was tested. Runcaciguat activates sGC in its haem-free configuration of NO (Figure 3A) [18]. This pharmacological activation of sGC provided additional information about its function. Runcaciguat was applied as a bolus at a maximal concentration of 10^−6^ mol L^−1^ for 10 min. It induced a faster response in juvenile vessels as compared to aged vessels, most prominently at 2 min post application (Figure 3B). At this time point, cGMP levels were lower in aged vessels (Figure 3C). Furthermore, the pharmacological inhibition of PDE5, which degrades cGMP, induced stronger relaxation in PE-pre-contracted juvenile vessels than in aged vessels (Figure 4A,B).

### 2.4. Vessel Histology Is Similar between Juvenile and Aged Mice

**Figure 5:**
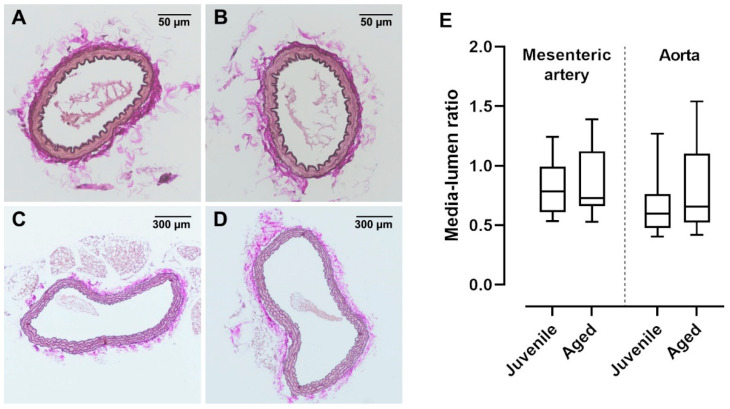
van Gieson-stained cross sections of mouse mesenteric artery and aorta. Vascular walls of both (**A**) juvenile and (**B**) aged mesenteric arteries showed no structural differences and had a similar number of elastic fibres. Neither (**C**) juvenile (*n* = 36) nor (**D**) aged (*n* = 19) aortae showed atherosclerotic alterations. (**E**) The media lumen ratios of juvenile mesenteric arteries (*n* = 26) and aortae (*n* = 20) were not significantly different from aged mesenteric arteries (*n* = 31) and aortae (*n* = 19), respectively (*p* > 0.05, Mann–Whitney test).

In old mice (>12 months), vessel dysfunction is accompanied by irreversible histological changes characterized by atherosclerotic plaques and vascular wall remodelling [19]. In the present study, a relatively early advanced life span was investigated. Interestingly, ACh-induced vasorelaxation was already reduced in aged mice (40 weeks) as compared to juvenile mice (13 weeks). Histological investigations of mesenteric arteries and aortae showed no visible changes in the vessel structure (Figure 5A–D). Furthermore, the media-to-lumen ratios of mesenteric arteries as well as aortae were not different between the aged and juvenile groups (Figure 5E).

### 2.5. Differential Expression of mRNA of sGC Subunits and PDE5

**Figure 6:**
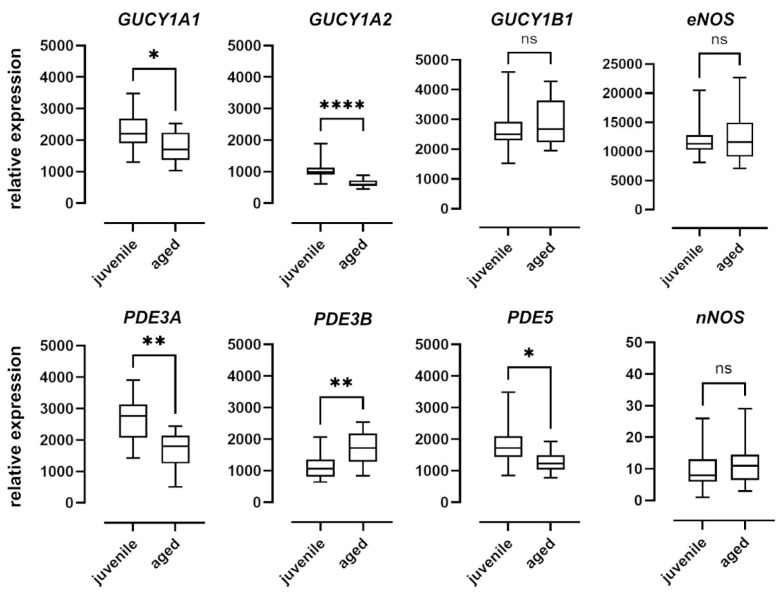
mRNA expression of sGC subunits and isoforms of PDE and NOS. Expression levels of the alpha subunits GUCY1A1 and GUCY1A2 of soluble guanylyl cyclase (sGC) were significantly higher in juvenile mesenteric arteries (*n* = 16) compared to aged vessels (*n* = 10). The expression of the beta subunit GUCY1B1, however, was not significantly different. Juvenile mesenteric arteries (*n* = 16) showed a significantly higher expression of PDE3A and PDE5 compared to aged vessels (*n* = 10); however, the expression of PDE3B was significantly lower. Expression levels of both eNOS and iNOS were not significantly different between the two groups (juvenile: *n* = 23, aged: *n* = 17). Data are represented as box and whisker plots of mRNA expression levels normalized to the housekeeping gene RPL32 (* *p* < 0.05, ** *p* < 0.01, **** *p* < 0.0001, Mann–Whitney test).

To examine the effect of aging on the NO–sGC pathway on a molecular level, we quantified the mRNA expression of the enzymes involved in the mesenteric arteries of juvenile and aged mice. Relative expression levels were determined using the ΔCt method and expressed as a ratio of the target gene’s expression level to that of the housekeeping gene (Figure 6). While there were no differences in the expression levels of nNOS and eNOS between the two groups, nNOS was expressed at extremely low levels in both groups. Expression of iNOS was below the detection level in both groups (data not shown). The α1 (GUCY1A1) and α2 (GUCY1A2) subunits of sGC were expressed at significantly lower levels in aged vessels as compared to juvenile vessels. The β1 subunit (GUCY1B1), however, was expressed at comparable levels in both groups. PDE5 expression levels in aged mice were also lower than in juvenile mice (Figure 6). PDE3A expression levels were lower and PDE3B levels were higher in aged as compared to juvenile vessels (Figure 6).

## 3. Discussion

In the present study, we demonstrated that the age-dependent decline in the relaxing ability of mesenteric resistance arteries of male mice is associated with an impaired sGC function. Aged vessels relaxed slower than juvenile arteries in response to the native dilatator NO and to the NO-independent sGC activator, runcaciguat. In addition, the mRNA-expression of two sGC alpha subunits was significantly lower in aged compared to juvenile vessels. The data suggest the important contribution of NO-related pathways in VSM cells to the impaired dilatory vessel function during aging.

A decrease in endothelium-dependent vasodilatation is a main and well-known feature of vessel aging, and it has been demonstrated in numerous studies in animals as well as in humans [3,4,5,6,7]. Defects in the NO pathway, deficits in EDHF-mediated responses, and changes in the function of the prostaglandin system contribute to endothelial dysfunction in the process of aging [3,20]. Endothelial dysfunction is characterized by reduced flow-mediated dilatation [21]. Furthermore, agonist-induced dilatation is impaired [22]. In the present study, ACh-induced vasorelaxation was reduced in aged animals as compared to young ones, in agreement with previous observations in rats and humans [4,15,23]. However, the non-selective inhibition of NOS using L-NAME did not only decrease the overall response of both juvenile and aged vessels to ACh, but also abolished the difference between the group responses. This strongly suggests the critical role of the NO system in the dilatation of small mesenteric arteries of C57BL6 mice and in the age-related reduction in the dilatory capacity of these vessels, which is in line with previous studies demonstrating impaired NO-mediated relaxation in large arteries, resistance vessels, and arterioles [24,25,26,27].

Endothelium-independent vasorelaxation was tested by the cumulative application of the NO donor, SNP. The concentration-dependent relaxations did not differ between juvenile and aged vessels, suggesting that NO signalling pathways were not impaired in VSM cells. In the literature, results regarding endothelium-independent relaxation are inconsistent. For example, endothelium-independent NO-mediated relaxation in three to four-month-old rats was similar as compared to 20-month-old rats [15], while it was reduced in 45-week-old rats as compared to 12-week-old rats [16]. These contrary observations may reflect different aging models. Furthermore, it cannot be excluded that differences in the nutrition, motor activity, and environmental conditions of the animals contribute to the inconsistent observations. For a more detailed investigation of NO signalling in VSM cells, endothelium-independent relaxation in response to the bolus application of NO was followed up for 10 min. This experimental design enabled a closer view of the dynamics of NO signalling in VSM cells. The experiment revealed prompt relaxation in juvenile vessels in contrast to sluggish relaxation in older vessels. More notably, this difference could be seen in the physiologically important range of up to 2 min. Several mechanisms of vascular adaptation to metabolic demands and vessel-based autoregulation of organ perfusion include NO signalling via sGC. Most of these dynamic adaptations of local and systemic circulation work within a time period that lasts from seconds to minutes; one example is the myogenic response [28]. The NO-system modulates this important mechanism for the control of organ perfusion. It has also been shown that NO release varies with the frequency of the fluctuations of blood pressure and blood flow, respectively, and dampens blood pressure variations in a low-frequency range. The latter may be an antihypertensive effect [29,30]. Thus, studying these dynamics is important for understanding age-related differences in cardiovascular control.

To support the assumption that the age difference in relaxation dynamics is related to a change in sGC activity, cGMP concentrations in the vessels were measured. For this purpose, the NO- and haem-independent sGC activator, runcaciguat, was applied as a bolus. Again, the relaxation was slower in aged vessels as compared to juvenile vessels. More importantly, the slower relaxation was accompanied by lower cGMP levels 2 min after runcaciguat was applied. Thus, both the natural as well as the pharmacological stimulation of sGC point to a decline in its activity with increasing age. The smaller effect of PDE5 inhibition in aged vs. juvenile mice is in agreement with this conclusion. However, the lower PDE5 mRNA expression in aged animals also has to be considered in this context.

sGC is a heterodimeric haemoprotein, consisting of an alpha and a beta subunit, each of which has two isoforms in vertebrates (α1, α2, and β1, β2) [31]. The α1/β1 heterodimer is probably most widely expressed in mammals. However, the α2/β1 variant has also been found in several tissues [32]. Thus, sGCα1/β1 and sGCα2/β1 isoforms seem to be the physiologically active heterodimers [33]. Mice lacking the α1 subunit of sGC show decreased ACh-induced vasodilation and are prone to hypertension, while the deletion of the β1 subunit in smooth muscle induces a complete loss of sGC function [34,35,36,37]. Experiments with mice deficient in either α1- or α2-sGC underscore the functional importance of both sGCα1/β1 and sGCα2/β1 heterodimers in aortic relaxation [38]. In mice, sGCα1/β1 is mainly responsible for the modulation of renal blood flow and systemic blood pressure. Interestingly, sGCα2/β1 can replace sGCα1/β1 function [37]. In the present study, mRNA for α1 (GUCY1A1), α2 (GUCY1A2), and β1 (GUCY1B1) was detected in resistance mesenteric vessels, with the lowest expression for GUCY1A2, which agrees with observations in the rat aorta [38]. GUCY1A1 and GUCY1A2 were expressed at lower levels in aged as compared to juvenile vessels, which might explain the decline in sGC function. The cGMP-degrading enzyme, PDE5, was also differentially expressed when juvenile and aged vessels were compared. We assumed a compensatory decrease in PDE5 due to reduced cGMP levels. In contrast to the findings of the present study, an upregulation of PDE5 has been observed in the iliac arteries of middle-aged rats and in senescent human VSM cells [39,40]. PDE5 expression seems not to be influenced by sGC, because sGCα1/β1-deficient mice show comparable PDE5 expression to that in wild-type mice [41]. We also observed a significant reduction in the mRNA expression of the functionally less significant PDE3A isoform in aged mice compared to juvenile mice. The activation of sGC in rat pulmonary artery smooth muscle cells upregulates the protein expression and activity of PDE3A, an effect reversed by sGC inhibitors [42]. A similar effect is seen in sGC knock-out mice, which have a 50% reduction in the expression and activity of PDE3A compared to that found in wild-type mice [43]. Taken together, GUCY1A1, GUCY1A2, PDE3A, and maybe PDE5 mRNA expression patterns correspond with the decline in the dynamics of sGC activity shown here and support the notion of altered NO signalling and cGMP metabolism, respectively, in old VSM cells. Protein expression data are not shown because the specificity of antibodies for the sGC subtypes and PDEs is lower than that for qPCR probes. Therefore, mRNA data may reflect gene expression better than protein expression data in this case. In order to exclude a possible significant contribution of eNOS to an age-related decrease in relaxation, its expression was analysed. Results revealed no difference in eNOS mRNA levels between groups. The nNOS expression was also investigated. It was expectedly very low and may not reach biological significance. The expression of iNOS was below the detection level, which suggests an absence of inflammatory processes in aged vessels and is in agreement with the histological finding that there is no atherosclerotic remodelling of aged vessels. The observation corresponds with the literature and reflects the comparatively lower contribution of NO from nNOS to vasorelaxation [44,45]. Studies on the effect of age on eNOS expression do not provide a uniform picture and report either unaltered or decreased eNOS mRNA and protein expressions [46,47,48,49,50]. Other studies have also found increased eNOS mRNA expression [50,51]. Although the majority of the results may point to the contribution of eNOS to a decreased endothelial relaxing function in old vs. young animals, further studies on the contribution of eNOS to vasorelaxation in general as well as with respect to age are warranted.

In conclusion, altered NO signalling in VSM impairs the age-related vasorelaxation of resistance arteries in male mice. Blunted sGC activity in the VSM compartment hallmarks vascular aging, which occurs early in life, in the absence of overt histopathological changes.

## 4. Materials and Methods

### 4.1. Experimental Animals

Male mice (C57BL/6) were maintained at the animal facility of the Charité—Universitätsmedizin Berlin under a 12 h light/dark cycle in enriched cages. Juvenile (age: 91–97 days ~13 weeks, *n* = 65) and aged (age: 280–288 days ~40 weeks, *n* = 45) were fed with standard pellet food sniff (ssniff-Spezialdiäten GmbH, Soest, Germany, sodium content: 0.24%) and allowed access to tap water ad libitum. 

### 4.2. Chemicals and Drugs

The drugs used in this experiment—acetylcholine (ACh), phenylephrine (PE), L-NAME (*N*Μ-nitro-L-arginine methylester hydrochloride)—were purchased from Sigma, St. Louis, MO, USA; SNP was purchased from Carl Roth, Karlsruhe, Germany; sildenafil citrate from BioVision, Milpitas, CA, USA. Runcaciguat was provided by Bayer AG, Research & Development, Pharmaceuticals (Wuppertal, Germany). All salts, glucose and HEPES used to prepare buffer solutions were purchased from Carl Roth, Karlsruhe, Germany. PE and ACh were dissolved in distilled water and stored at −20 °C. The SNP solution was prepared in distilled water immediately before use and stored in an amber tube. L-NAME was dissolved using ultrasound in distilled water. Runcaciguat was initially prepared in dimethylsulphoxide (DMSO, Thermo Scientific, Bellefonte, PA, USA) and stored at −20 °C.

### 4.3. Vessel Function

Mesenteric arteries with the size of resistance arteries (diameter of non-pressurized arteries: 59–67 µm) were used in the present study. To isolate the vessels, the mice were killed by cervical dislocation under isoflurane. The intestines were removed and placed in ice-cold and oxygenated buffer (146 mmol L^−1^ NaCl, 4.5 mmol L^−1^ KCl, 1.2 mmol L^−1^ NaH_2_PO_4_·2H_2_O, 1 mmol L^−1^ MgSO_4_·7H_2_O, 5.5 mmol L^−1^ glucose, 0.025 mmol L^−1^ Na (EDTA), 5 mmol L^−1^ HEPES, and 1.6 mmol L^−1^ CaCl_2_·2H_2_O; pH 7.4). The mesenteric arterial network was isolated from the tissue and 2 mm long segments in the size of resistance arteries were cut. The segments were then mounted onto two stainless steel wires (diameter: 40 μm) in a small vessel myograph (model 410A, Danish Myo Technology A/S, Hinnerup, Denmark), under isometric conditions. The vessel segments were allowed to equilibrate in carbogenated (95% O_2_, 5% CO_2_) experimental solution (119 mmol L^−1^ NaCl, 4.7 mmol L^−1^ KCl, 1.2 mmol L^−1^ KH_2_PO_4_, 1.2 mmol L^−1^ MgSO_4_·7H2O, 6.1 mmol L^−1^ glucose, 25 mmol L^−1^ NaHCO_3_, and 2.5 mmol L^−1^ CaCl_2_·2H_2_O; pH 7.4) at 37 °C. The resting tension of the arteries was set according to Mulvany’s normalization procedure [52]. The diameter of the vessel was set to 90% of that calculated for a transmural tension of 100 mm Hg, as described by Mulvany and Halpern [53].

To test the activity of the mounted and equilibrated vessels, the chamber solution was changed to K-PSS (123.7 mmol L^−1^ KCl, 1.2mmol L^−1^ KH_2_PO_4_, 1.2 mmol L^−1^ MgSO_4_·7H_2_O, 6.1 mmol L^−1^ glucose, 25 mmol L^−1^ NaHCO_3_, and 2.5 mmol L^−1^ CaCl_2_·2H_2_O; pH 7.4). After maximum contraction was reached, the K-PSS was washed out and replaced with an experimental solution. To measure the relaxing ability of arteries, they were pre-contracted with phenylephrine (PE, 10^−5^ mol L^−1^) or 60mM KCl (63.7 mmol L^−1^ NaCl, 60 mmol L^−1^ KCl, 1.2 mmol L^−1^ KH_2_PO4, 25 mmol L^−1^ NaHCO_3,_ 1.2 mmol L^−1^ MgSO_4_·7H_2_O, 11.1 mmol L^−1^ glucose, 0.026 mmol L^−1^ Na (EDTA), and 1.6 mmol L^−1^ CaCl_2_·2H_2_O). Endothelium-dependent relaxation was measured by applying ACh in cumulative concentrations (10^−9^~10^−6^ mol L^−1^, each concentration for 50 s), and endothelium-independent relaxation was measured by the application of sodium nitroprusside (SNP, 10^−11^~10^−5^ mol L^−1^, each concentration for 30 s). Vessels were pre-incubated with L-NAME (10^−4^ mol L^−1^) in the chamber for 30 min to estimate the contribution of the NO system. The PDE5 inhibitor sildenafil (10^−9^~10^−6^ mol L^−1^_,_ each concentration for 30 s) was used to characterize the NO–sGC–cGMP axis. The NO- and haem-independent sGC function was tested using the sGC activator, runcaciguat (10^−6^ mol L^−1^).

### 4.4. *Direct cGMP ELISA*

Mesenteric arteries were isolated. After normalization and KCl-testing, arteries were incubated with L-NAME (10^−4^ mol L^−1^) for 30 min and pre-contracted with PE (10^−5^ mol L^−1^). Then, runcaciguat (10^−6^ mol L^−1^) was applied as bolus. After 2 min, the arteries were shock-frozen and stored at −80 °C until cGMP concentration was measured using a direct cGMP ELISA kit (Enzo life Science, Lausen, Switzerland) according to the manufacturer’s instructions.

### 4.5. Gene Expression Analysis

Isolated mesenteric arteries were frozen and stored at −80 °C. RNA was isolated with RNA-Bee-reagent (Biozol, Eching, Germany) and reverse transcribed with the High-Capacity cDNA RT Kit (Applied Biosystems, Foster City, CA, USA) according to the manufacturer’s protocols. qPCR was performed using a Lightcycler LC480 (Roche Diagnostics, Mannheim, Germany) according to the manufacturer’s protocol using the hydrolysis probe fluorescent detection of the DNA generated during PCR. The expression levels of mRNA were normalized to the housekeeping gene RPL32 (primer sequences: see Table 1) and relative expression levels were calculated by ΔCt method.

### 4.6. Morphometric Analysis

Aortae (thoracic and abdominal) and small branches of mesenteric arteries were dissected and placed in 4% buffered formalin (SAV Liquid Production GmbH, Flintsbach, Germany) for fixation. The fixed vessels were embedded in paraffin, sliced (4 μm for aorta and 6 μm for mesenteric arteries), and stained using van Gieson’s solution (1% acid fuchsin and saturated aqueous solution of picric acid mixed 1:10) for elastic fibres. Image J 1.48 was used to measure arterial dimensions and to calculate the media lumen ratio (digital images: Color Camera Nikon DS-Ri2 Numerical Aperture) [54].

### 4.7. *Statistics*

Mean, standard error of the mean (SEM), and EC50 were calculated using GraphPad Prism 9.0.1 (GraphPad software, San Diego, CA, USA). Data for time and concentration-dependent vessel responses are presented as mean ± SEM. Data were tested for normal distribution using the Shapiro–Wilk test. However, most data sets were not normally distributed. Therefore, non-parametric tests were used for all of the comparisons. Time and concentration-dependent differences between groups were tested by the Brunner test, a non-parametric counterpart of the two-way ANOVA, which tests the hypotheses of a global difference between the two groups and a global effect of change [55]. The Mann–Whitney U test (GraphPad Prism 9.0.1) was used post hoc to analyse the differences between the vessel responses of the two groups at individual time points and concentrations, as well as the EC50. Morphometry and mRNA expression data are presented as median, box, and whiskers (min., max.), and differences were tested using the Mann–Whitney U test.

## Figures and Tables

**Table 1:** Sequences of primers and probes used for gene expression analysis.

Gene	Forward Primer (5′–3′)	Reverse Primer (5′–3′)	TaqMan Probe (5′–3′)
nNOS	CCCGACAGGCCAAAGAAATA	ACGTCCCCGCAGACATAAAT	GCTGGCCGAGTCTGTGTACCGCGCCC
iNOS	AGCGGCTCCATGACTCCCA	GGCACCCAAACACCAAGCT	ATGCGGCCTCCTTTGAGCCCTTT
eNOS	GACCCTCACCGCTACAACAT	ATGAGGTTGTCCTGGTGTCC	AGGATGTGGCTGTGTGCATGGATCT
GUCY1a1	ATTTCATGCTGGACCGAGAC	TTCCCTTGGAAGTCCCTCTT	TAACGGCATCAGAAGGCTGGTGAAC
GUCY1a2	CTGGACTCACTAGGCGAAAG	GTCATGTGTATCGTCTGAGGC	CAGCCTCCTGACGGCGCCCTT
GUCY1b1	AGCCCTTACACCTTCTGCAA	CATTGCCACACTGAGTGACC	CCTTTTCACATCATATTTGACCGGAACC
GUCY1b2	CAGGTGTTGTGGGAGACAAG	TCCTAGAGGCCGTGTTTACG	CCCGGTACTGCCTGTTTGGTGACAC
PDE5	CCGACTTCAGCTTCAGTGACTT	GGTCAGTGAACATCCGAATTG	TGTCTGATCTGGAAACAGCGCTGTG
RPL32	TTCATCAGGCACCAGTCAGA	TTGTCAATGCCTCTGGGTTT	TGTGAAAATTAAGCGAAACTGGCGG

## Data Availability

The data presented in the study are contained within the article.
